# ‘teen Mental Health First Aid’: a description of the program and an initial evaluation

**DOI:** 10.1186/s13033-016-0034-1

**Published:** 2016-01-19

**Authors:** Laura M. Hart, Robert J. Mason, Claire M. Kelly, Stefan Cvetkovski, Anthony F. Jorm

**Affiliations:** Population Mental Health Group, Centre for Mental Health, Melbourne School of Population and Global Health, University of Melbourne, Melbourne, Australia; School of Psychology and Public Health, La Trobe University, Melbourne, Australia; Mental Health First Aid, Melbourne, Australia; School of Psychology, Deakin University, Geelong, Australia

**Keywords:** Mental Health First Aid, Mental health literacy, Stigma, Adolescents, Secondary school

## Abstract

**Background:**

Many adolescents have poor mental health literacy, stigmatising attitudes towards people with mental illness, and lack skills in providing optimal Mental Health First Aid to peers. These could be improved with training to facilitate better social support and increase appropriate help-seeking among adolescents with emerging mental health problems. *teen Mental Health First Aid* (teen MHFA), a new initiative of Mental Health First Aid International, is a 3 × 75 min classroom based training program for students aged 15–18 years.

**Methods:**

An uncontrolled pilot of the teen MHFA course was undertaken to examine the feasibility of providing the program in Australian secondary schools, to test relevant measures of student knowledge, attitudes and behaviours, and to provide initial evidence of program effects.

**Results:**

Across four schools, 988 students received the teen MHFA program. 520 students with a mean age of 16 years completed the baseline questionnaire, 345 completed the post-test and 241 completed the three-month follow-up. Statistically significant improvements were found in mental health literacy, confidence in providing Mental Health First Aid to a peer, help-seeking intentions and student mental health, while stigmatising attitudes significantly reduced.

**Conclusions:**

teen MHFA appears to be an effective and feasible program for training high school students in Mental Health First Aid techniques. Further research is required with a randomized controlled design to elucidate the causal role of the program in the changes observed.

**Electronic supplementary material:**

The online version of this article (doi:10.1186/s13033-016-0034-1) contains supplementary material, which is available to authorized users.

## Background

*Mental Health First Aid* is defined as the help provided to a person developing a mental health problem or experiencing a mental health crisis. The first aid is given until the appropriate professional treatment is received, or the crisis resolves [1]. Mental Health First Aid techniques have been taught in training programs offered by Mental Health First Aid (MHFA) since 2001. A recent meta-analysis of 15 separate evaluations of MHFA training found that it is effective in improving knowledge, attitudes and behaviours related to mental ill-health. The analyses found small to medium effect sizes, with the largest gains seen in improvements to knowledge [2]. By 2011, the MHFA training program had been presented to more than 1 % of Australian adults and had spread to 17 countries outside its headquarters in Australia [3]. It has also been recognised with a number of awards for excellence, is listed in the US Substance Abuse and Mental Health Services Administration’s (SAMHSA) National Registry of Evidence-Based Programs and Practices (http://www.nrepp.samhsa.gov/ViewIntervention.aspx?id=321) and has been cited as a model of ‘radical efficiency’ in the provision of social services [4].

Although the original version of the training was designed to teach adults how to assist other adults with mental health problems, it soon became clear that tailoring for other specific cultural and age groups was required. Versions of the MHFA course have now been developed for Aboriginal and Torres Strait Islander peoples [5, 6], Chinese Australians [7] and for adults and professionals in contact with young people with emerging mental illness (e.g. the Youth Mental Health First Aid program [2, 8, 9]). Yet while there are current, established and effective programs for improving MHFA skills among adults, there are no corresponding programs designed to teach adolescents how to assist their peers. Similarly, although there are existing school-based mental health literacy programs [10], these do not focus specifically on MHFA knowledge and skills.

### The case for training adolescents

Adolescence is the peak period for the onset of mental illness. Half of all people who will ever experience a mental illness in their lifetime will have had their first episode by age 18 [11]. It is estimated that one in four 16–24 year olds will experience a mental illness in any 12-month period [12]. Because of the important social, emotional and physical developmental goals that are achieved in this life stage, the onset of mental illness can be particularly debilitating and can lead to life-long burden and disability [13]. The provision of MHFA to adolescents is clearly needed. This is ideally provided by adults, who have a greater capacity to solve problems, take on caring responsibilities and provide practical support. However, provision of MHFA by adults is not sufficient, because adolescents often report a preference for seeking help from peers [14]. Given that adolescents are being called upon to provide support for peers with mental health problems, they need to be adequately equipped for this role. Yet it is clear that many adolescents have poor mental health literacy, stigmatising attitudes towards people with mental illness, and lack the specific knowledge and skills required to provide social support and prompt appropriate help-seeking.

### Adolescents have poor mental health literacy

*Mental health literacy* has been defined as “knowledge and beliefs about mental disorders which aid their recognition, management or prevention” [15]. For young people, the International Declaration on Youth Mental Health has set the objective of mental health literacy as “Raise awareness among young people, families and communities of the determinants of mental health and the mental health needs of young people aged 12–25 years” [16]. Mental health literacy includes: the ability to recognise specific disorders, knowing how to seek information about mental health, an understanding of risk factors and causes, as well as knowledge and attitudes that promote appropriate professional help-seeking and engagement in suitable self-help treatments [17]. Young people are often fearful of professional treatment and some view consultation with mental health experts as an option of last resort [18, 19]. In addition, there are some problems that young people feel particularly reluctant to tell an adult about. Studies investigating disclosure of suicidal thoughts reveal that most young people will choose to talk to a suicidal peer on their own, rather than enlist the help of a responsible adult. Of concern is the finding that those who are most vulnerable are the least likely to disclose suicidal thoughts to an adult; if a young person has personal experience of suicidal thoughts and behaviours, they are less likely to tell an adult about a peer’s disclosure of suicidality [20].

Only a minority of young people with clinically significant symptoms of mental illness ever seek appropriate professional help [21, 22]. This occurs despite early intervention for adolescents with mental illness being particularly important. In 15–25 year-olds, early intervention is thought to: increase the likelihood that developmental goals will be achieved, potentially arrest the progression of illness, and increase the quality of life in those with established mental illness, even where pathology remains unaffected by treatment interventions [23, 24].

Beliefs about the role of adults and mental health professionals in being able to help young people with mental illness therefore need to be improved, in order for young people to facilitate early intervention in their peers or to seek appropriate help for themselves.

### Adolescents have stigmatising attitudes towards people with mental illness

Stigmatising attitudes towards people with mental illness are a significant barrier to help-seeking and social support [25]. In 2005 a national telephone survey, of 3746 Australians aged 12–25 years, found that attributing mental disorder to a personal weakness rather than an illness was associated with less intention to seek help from a doctor and less positive beliefs about professional sources (including doctors, counsellors, and psychologists) [26]. Furthermore, greater scores on the social distance scale were associated with less intention to seek help [26].

Similar studies have also found that adolescents score higher on measures of desired social distance from individuals with mental illness, and are more likely to believe that mental illness is a personal weakness, than adults [27]. Young people’s stigmatising attitudes towards those with mental illness have also been found to be associated with rates of help-seeking [19], problem recognition or labelling [28] and MHFA behaviours, such as assessing for suicide risk [29].

Importantly, many researchers have suggested that interventions designed to reduce young people’s stigmatising attitudes would improve help-seeking intentions and increase the quality of first aid behaviours provided to peers [29–31]. Furthermore, there is evidence to suggest that exposure to public health campaigns about mental illness is associated with reductions in beliefs that individuals with mental illness are `weak not sick’ [26]. So there is much to be gained from educating adolescents about mental illness and the impact that stigmatising beliefs can have on their ability to support or seek help for a peer with a mental health problem.

### Improving Mental Health First Aid behaviours to facilitate social support and help seeking

Adolescents have been consistently found to prefer disclosing symptoms of mental illness to their peers and are reluctant to seek adult intervention or professional help [22, 32, 33]. Importantly, despite the strong preference for seeking help from friends, adolescents are not well equipped to cope with a friend’s disclosure of a mental health problem [18, 32, 34]. In a large longitudinal survey, it was found that only 15 % of 534 respondents aged 12–25 years who reported trying to help someone with a mental health problem in the last 12 months, actually reported encouraging the person to seek professional help, and only 3 % reported telling someone else (such as an adult) about the person’s problem [34]. Other research has found that young people are also less likely to ask about suicidal thoughts in a peer [30].

Nevertheless, the peer group of a young person with a mental illness can be a source of great support, comfort and information [22]. Help-seeking studies in young people suggest that the decision to seek help, to engage in appropriate treatments and adhere to its course, are all heavily influenced by the attitudes and suggestions of the social network or peer group [14, 33, 35]. Young people’s knowledge of how to support someone with a mental illness to seek out appropriate help is therefore a potential avenue for increasing early intervention and reducing untreated mental illness in young people.

### teen Mental Health First Aid

teen MHFA is a new initiative of the MHFA program. teen MHFA involves the delivery of a short course to adolescents in years 10–12 of secondary school, or roughly between 16–18 years. It uses age-appropriate materials developed from research with experts and consumers in the field of youth mental health [36], consultation with the education sector, and findings from research on health behaviour change [37–40] and barriers to adolescent help-seeking [19, 22, 32]. The program focuses on developing knowledge and skills in: recognising warning signs that a peer is developing a mental health problem, understanding how to talk to a peer about mental health and seeking help, when and how to tell a responsible adult, where to find appropriate and helpful resources about mental illness and professional help, and how to respond in a crisis situation. The teen MHFA program aims to increase mental health literacy, decrease stigmatising attitudes towards individuals with mental illness, and improve MHFA behaviours.

The aim of the current study was to conduct an uncontrolled pilot evaluation of teen MHFA to provide initial evidence of program efficacy, test relevant measures of student knowledge, attitudes and behaviours, and to examine the feasibility of providing the program in Australian secondary schools.

## Methods

### Participants

Eligible students were recruited from host schools agreeing to participate in the evaluation research. These were government, Catholic or independent secondary schools in the greater Melbourne area of Australia. Schools were eligible if they were willing to withhold other mental health training until the completion of the research (at the conclusion of the 3 month follow-up period), or if they had not provided similar mental health literacy programs to their year 10–12 students in the last 12 months. Four schools agreed to host the study: one metropolitan government school, one provincial government school, one metropolitan independent school, and one provincial Catholic school. Details of the sociodemographic characteristics of the four schools are given in Additional file 1. In return for hosting the research, schools were offered teen MHFA training to all students in years 10 and 11 free of charge, in addition to at least one Youth MHFA course for parents and one for teachers of students attending the teen training. More courses were provided where warranted by parent and teacher interest.

All students at participating schools in years 10 and 11 (aged 15–17 years) were offered the teen MHFA training program, whether or not they participated in the evaluation research. Teachers and parents of student participants were also invited to provide feedback in survey format. All intervention and survey administration sessions were conducted between February and November 2013 (in Australia, school years run between February and December). The terms when the intervention was run was not standardized across schools.

### Intervention

The teen MHFA training intervention involved three 75-minute classroom sessions facilitated by an accredited MHFA Instructor with specific training and experience in youth mental health. Sessions were presented to class groups of 15–25 students during normal school hours. All students attended the three training sessions, as the course was imbedded within their regular curriculum. For students who did not have parental consent, or did not provide assent to participate in the research, they were given alternative activities to do in class by the regular classroom teacher, or allowed private study time at a location outside the classroom (e.g. in the school library). The students’ regular classroom teacher was present during the training. Training was normally completed within 5–8 school days, depending on timetabling at each school, with at least 1 day between each session. The training involved: a didactic powerpoint presentation; video presentations, role-plays, group discussion and small group activities. A student booklet was provided for each participant, for use in sessions and for reference after course completion [41]. A teaching manual was provided to instructors to guide facilitation and ensure fidelity and consistency.

**Fig. 1:**
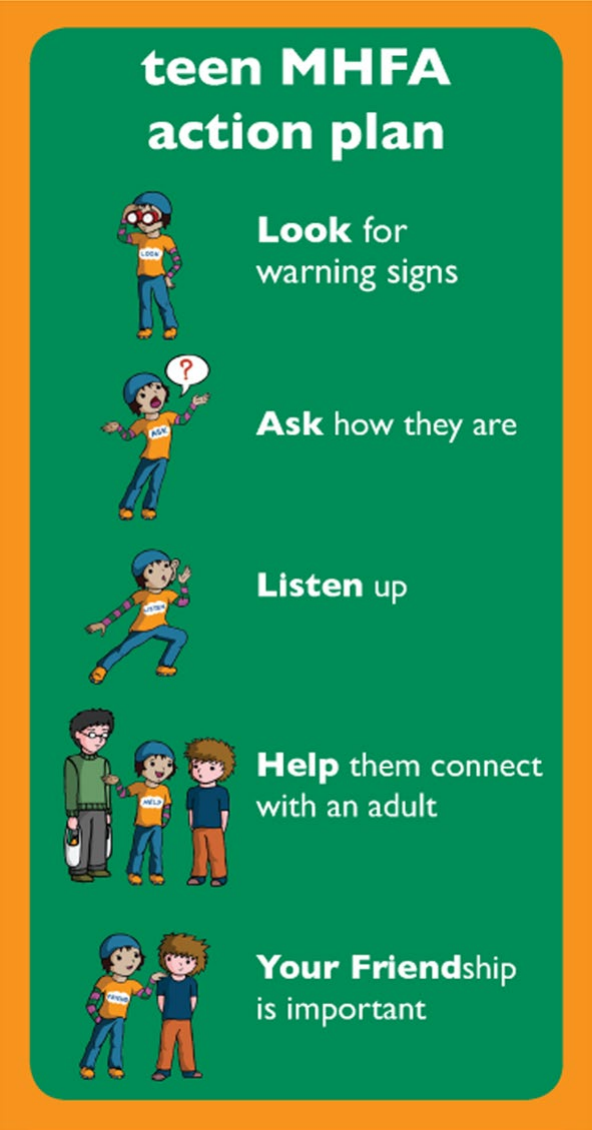
teen Mental Health First Aid action plan. The central teaching of teen Mental Health First Aid training is a five-point action plan, developed from the outcomes of a Delphi study [36]. It is designed to communicate five first aid strategies in an easy to remember format; once each action has been explained and discussed in detail throughout the course, the action plan is then referred to in short as “Look, Ask, Listen, Help, Your Friend”

Program content is outlined in Table 1. The central teaching of MHFA training programs is an action plan. Modelled on the action plans developed for MHFA training courses for adults [8, 42] and based on the key messages for adolescents from a Delphi expert consensus study [36], the teen MHFA action plan provides five first aid strategies taught in a mnemonic designed to be easy to remember (see Fig. 1). All materials and program content were piloted with two groups of adolescents (n = 23), whose feedback on how to improve the training was incorporated before the uncontrolled evaluation began.

**Table 1:** Structure and content of the teen Mental Health First Aid training

Session 1: 75 min	Session 2: 75 min	Session 3: 75 min
Topics presented: What is mental health? What are mental health problems? Types of mental health problems Impact on young people Stigma Appropriate help	Topics presented: Helping a friend in a mental health crisis What is mental health first aid? What is a mental health crisis? Using the teen MHFA action plan to help a friend in crisis Recovery position	Topics presented: Helping a friend who is developing a mental health problem Importance of acting early Using the teen MHFA action plan to help a friend developing a mental health problem Helpful links and resources
Videos: * Talking about it* *1* (4:50 s) * Getting help* (5:32)	Video: *Mates* (13:55)	Videos: * Talking about it* *2* (4:14) * Talking about it* *3* (6:02)
Activities: Group discussion of how mental health problems impact on young people Identifying supportive adults Relaxation	Activities Group discussion of confidentiality vs safety Role play recovery position	Activities: Group discussion of Luke and Ali’s stories Role play using the action plan

Because a core message of the teen MHFA training is to seek assistance from a trusted and reliable adult when a peer is experiencing a mental health problem, the Youth MHFA course was also provided to staff and parents at participating schools. One course was provided for teachers and another for parents. This ensured that adults who were called upon to assist adolescents were confident in providing support and could facilitate appropriate referral pathways to effective treatment interventions.

### Procedure

Schools were initially approached to determine interest, before a memorandum of understanding was established between the research team and the school principal. Three weeks before the teaching sessions for students were due to begin, a plain language statement and consent form was sent to the parents of each student to receive the training, either via email or in hard copy via mail. Students completed the baseline survey up to 1 week before participating in the first session. The student surveys were administered online by surveymonkey.com. Students were sent a generic link to the survey by their school administrator. Survey sessions were scheduled during regular classroom time, where students either used their own laptop or a laboratory computer to access the link sent. Students provided their student ID as a unique identifier so that each of the three questionnaires could be paired at the completion of data-entry. If students had difficulty with their computer or the electronic link to the survey, they completed a hardcopy survey and this was handed to the regular classroom teacher, who passed it back to the research team for anonymous data entry. Completion of the measures took approximately 40 min.

The three sessions of the intervention were held across 5–8 school days, depending on timetabling at each school, with at least 1 day between each session. The post-course survey was then completed within 1 week of the final session.

### Measures

A survey questionnaire was developed to measure mental health literacy, stigmatising attitudes, MHFA behaviours, and the mental health and help-seeking status of adolescents. It was administered at three time points: before, immediately after, and 3 months after the training. The questionnaire included items adapted from the Australian National Survey of Youth Mental Health Literacy [43], which related to two vignettes; one depicting an adolescent experiencing depression with suicidal ideation (John), and another experiencing social phobia (Jeanie). The vignettes are provided in Additional file 2. All open-ended responses were coded by an independent researcher (Alyssia Rossetto) according to a structured protocol described in a separate publication [44] and blind to measurement condition. For some of the measures below, one-month retest reliability data is available for 165 students from a school which was not involved in this evaluation and did not receive the intervention.

#### Mental health literacy

*Problem recognition* was assessed by asking students to identify what, if anything, was wrong with the person in the vignette. Responses were open-ended. The labels given to these vignettes have been previously validated against the diagnoses of mental health professionals [45]. Furthermore, labelling a vignette accurately has been found to predict a preference for sources of help recommended by mental health professionals [46] and with better quality MHFA responses [47]. *Beliefs about help* were assessed by asking participants to rate a range of potential sources of help as likely to be helpful, harmful or neither, for the person in each vignette. Potential sources of help included: close friend, counsellor, family member, GP, minister/priest, parent, psychologist, school counsellor and teacher. These items were used to measure belief in getting adult help, which is a key message of the training [36].

#### Stigmatising attitudes

Students were asked to respond to seven questions assessing personal stigma towards the person in the vignette, on a Likert scale (1 = ‘strongly disagree’ to 5 = ‘strongly agree’). The questions were: (1) (John/Jeanie) could snap out of it if (he/she) wanted; (2) (John/Jeanie)’s problem is a sign of personal weakness; (3) (John/Jeanie)’s problem is not a real medical illness; (4) (John/Jeanie) is dangerous to others; (5) It is best to avoid (John/Jeanie) so that you don’t develop this problem yourself; (6) (John/Jeanie)’s problem makes (him/her) unpredictable; (7) If I had a problem like (John/Jeanie)’s I would not tell anyone. Five items adapted for young people from the Social Distance Scale [26, 48] asked whether the participant would be happy to: (1) develop a close friendship with (John/Jeanie); (2) Go out with (John/Jeanie) on the weekend; (3) go to (John/Jeanie)’s house; (4) invite (John/Jeanie) around to your house; (5) work on a project with (John/Jeanie). Each question was rated on a 4-point Likert scale (1 = ‘yes definitely’ to 4 = ‘definitely not’). The personal stigma and social distance items were used in combination to construct four stigma scales, which have previously been validated by exploratory structural equation modelling: *(1) weak*-*not*-*sick, (2) dangerous/unpredictable, (3) would not tell anyone,and (4) social distance* [49]. Retest reliabilities were respectively 0.79, 0.68, 0.60 and 0.77.

#### Mental Health First Aid intentions and behaviours

*Confidence* with providing first aid was assessed by asking how confident (5-point Likert scale) students felt in helping the person in the vignette, and this was considered the primary outcome of interest. Retest reliability for the confidence scores summed across the two vignettes was 0.67.

*Mental Health First Aid intentions* were assessed by asking: “If (John/Jeanie) was someone you knew and cared about, what would you do to help (him/her)?”. Open-ended responses were scored against the teen MHFA action plan taught in the course [44]. Previous research has shown that quality of MHFA intentions predicts quality of subsequent actions in young people [50].

Students’ *first aid experiences* were assessed at baseline by asking if in the last 3 months they had contact with anyone who they thought might have a mental health problem or experienced a mental health crisis. A mental health problem was defined as “a major change in a person’s normal way of thinking, feeling or behaving, which interferes with the person’s ability to get on with life, and does not go away quickly or lasts longer than normal emotions or reactions would be expected to”. The survey stated that this might involve a diagnosed mental illness, a worsening of mental health, an undiagnosed problem, or a drug or alcohol problem [41]. A mental health crisis was defined as when “a person is at increased risk of harm to themselves or to others”. The survey stated that crisis situations might include having thoughts of suicide, engaging in self-injury, being very intoxicated with alcohol or other drugs, or experiencing bullying or abuse. If they had had contact, the student was asked whether they had offered any help (4-point Likert scale from no help offered to a lot of help offered) and an open-ended question about what type of help it was.

Students’ *first aid experiences* were also assessed in more detail in the follow-up survey, using a modified First Aid Experiences Questionnaire [51], previously employed in evaluations of MHFA [52, 53]. The questionnaire asked: “Since completing the program have you come across someone you thought might have a mental health problem or has experienced a mental health crisis?” (yes/no), “What was the person’s relationship to you?” (open-ended), “Could you tell us something about the situation(s) and the problem(s) you believed the person was experiencing?” (open-ended), “Did you try to help this person?” (yes/no). For those who reported helping the person, the questions ran as follows: “Can you give any examples of how you tried to help the person?” (open-ended), “When assisting the person did you use the information provided in the program?” (no, not sure, yes), “Do you think what you did helped the person?” (5-point Likert scale from Yes, very helpful, to No, very unhelpful), “Would you like to comment on what happened?” (open-ended), “Do you think the information in the program contributed to how helpful you were in assisting the person”? (5-point Likert scale from Yes, definitely, to Definitely not), “When assisting the person, did you do anything differently from what you would have done before attending the program?” (no, not sure, yes) “Did you suggest to the person you were helping that they should talk to an adult about their problem?” (no, not sure, already being helped by an adult, yes), “As a result of your suggestion, did the person you were helping talk to an adult?” (no, not sure, N/A, yes). Those who reported not helping the person were asked for a reason(s) that they were not able to provide help.

#### Mental health and help-seeking status

*Student mental health* was assessed in the baseline and follow-up surveys with the K6 [54, 55]. The K6 is a measure of psychological distress with possible scores ranging from 6 to 30, which has been validated against clinical diagnosis. Although the student’s own mental health was not the focus of the course, this measure was included to examine whether the program might be associated with any iatrogenic effects.

Students’ *help*-*seeking intentions* were assessed by asking what the student would do if they had a problem like John/Jeannie. Responses were open-ended and coded into three categories, the frequencies of which were assessed over the three measurement time points. The *Talk tell ask* category included any responses mentioning “talking”, “telling” or “asking” someone about the problem. The *seek help* category included any responses in which students mentioned help-seeking actions. These included any responses suggesting participants would engage self-help activities (e.g. “Improve my diet”, “Get more sleep” or “Spend more time with friends”), help-seeking from an adult (e.g., “seek counselling”, “talk to family/family member/teacher/parent/GP”, “get medical help”), or some general notion of seeking help (e.g., “get help”, “seek help”). Finally, the *non help* category represented responses which made no mention of help-seeking actions (e.g. “would do nothing”, “sleep it off”), actively eluded help (e.g., “push people away”, “not tell anyone”) or were indicative of indecision (e.g. “uncertain”, “don’t know”). It was possible for student responses to be coded into more than one category.

In addition, students’ *self*-*report of mental health problems or crises* were assessed by asking students at baseline and at follow-up whether they had experienced a mental health problem or mental health crisis in the last 3 months, whether or not this had been diagnosed by a mental health professional. If a student said ‘yes’, they were then prompted to answer further questions about their *self*-*report of help received.* One question asked whether or not someone who was not a health professional (i.e., teacher, parent or friend) had tried to assist them with their problem or crisis, and what that person did to help. A second question asked whether they had received any treatment or advice from a professional specifically for their problem or crisis.

#### Participant satisfaction

In line with previous evaluations of MHFA training [52], satisfaction with the course was assessed immediately after the training with multiple-choice questions about presentation, materials and content. Additionally, a series of open-ended questions were presented regarding the strengths and weaknesses of the program, and how it could be improved in the future.

Parents and teachers of student participants also completed a questionnaire 3 months after course completion. This was designed to qualitatively examine their perceptions of the course and students’ training experience. A table summarizing results is shown in Additional file 3.

### Data analysis

#### Statistical analysis

Descriptive statistics, means and percentages, were used to examine the distributions of student demographic characteristics. To evaluate the effect of the training, changes in key outcomes over time (pre, post and follow-up) were examined using logistic and linear regression mixed-models for binary and continuous outcomes [56]. All results are reported as odds ratios (OR) or unstandardized regression coefficients (B), with 95 % confidence intervals (95 % CIs) and standard errors (SE) respectively. For continuous outcomes, outliers were removed for the data analysis. The strength of these maximum likelihood-based models is that they can account for the clustered data (i.e. the correlation of individual responses over time and the correlation of individual responses within schools).

Missing data were handled using uncongenial, multivariate imputation chained equations and were assumed to be missing at random (MAR) [57, 58]. A series of logistic regression models revealed that there were three covariates that significantly predicted drop-out from the study (p < 0.05): school (regional Catholic school students were less likely to drop-out, Metropolitan government school students more likely to drop-out and regional government school students had a similar drop-out rate to the comparison school, the independent metropolitan school); students who self-reported a mental health problem (more likely to drop-out); and whether students thought a family member would be helpful for the depression with suicidal thoughts (John) vignette (those who responded “yes” were less likely to drop-out). These covariates along with gender and the covariates of interest in the substantive analyses were included in the conditional imputation models, which accounted for the measurement level of the variables imputed (e.g. logistic and linear regressions for binary and continuous outcomes respectively). The inclusion of these predictors of drop-out lends greater plausibility to the MAR assumption [59]. In addition, all multiple imputations were conducted in wide dataset format to take into account the dependence of student responses over time and then reshaped into long format for the mixed-model analyses. Thirty imputed datasets were generated for each substantive analysis.

A repeated measures ANOVA also assessed change over time in the mean number of words used by participants in text-based responses to open-ended questions. All multiple imputations and substantive analyses were conducted using Stata IC/13.1.

#### Content analysis of open-ended responses

Inductive category development [60] was used to code open-ended responses into categories which could then be used to assess frequencies and analysed quantitatively. To do this, an independent researcher (Alyssia Rossetto) who was blind to measurement occasion scanned all open-ended responses for frequently occurring words or phrases. Category labels that were able to group similar data were drafted. A sample of responses and the proposed categories were then presented to the whole research team for discussion, and a codebook with inclusion and exclusion criteria for each category, was developed (available upon request). Response frequencies were then calculated for each category and analysed.

There were two exceptions to this process. First, the MHFA intentions and actual help given responses were coded according to an a priori categorisation scheme based on the teen MHFA action plan, as described previously [44]. Second, the *first aid experiences questionnaire* [51], completed at follow-up only, consisted of a mix of forced-choice and open-ended responses. Although the forced-choice responses provided frequency data, the open-ended responses were designed to elicit narrative data about first aid situations encountered. No analyses were conducted on these data; instead a selection of responses are presented to highlight the experiences of participants.

### Ethics, consent and permissions

Approval for the research was granted by the University of Melbourne Human Research Ethics Committee. Approval was also granted by the Victorian Department of Education and Early Childhood Development, and the Catholic Education Office Melbourne. To be eligible to participate in the evaluation research, students were required to have parental consent, provide student assent before completing surveys, and plan to attend the teen MHFA training. Students with a known current mental health problem, previous experience of mental illness or suicide bereavement were encouraged to speak to their mental health professional, school counsellor and/or parents before deciding whether to participate.

## Results

### Student demographics

The student sample at baseline was aged between 14 and 17 years (*M* = 15.98, *SD* = 0.76). Forty-nine percent were female, 32 % were in year 10, and 7 % from a non-English-speaking background.

### Participant flow

**Fig. 2:**
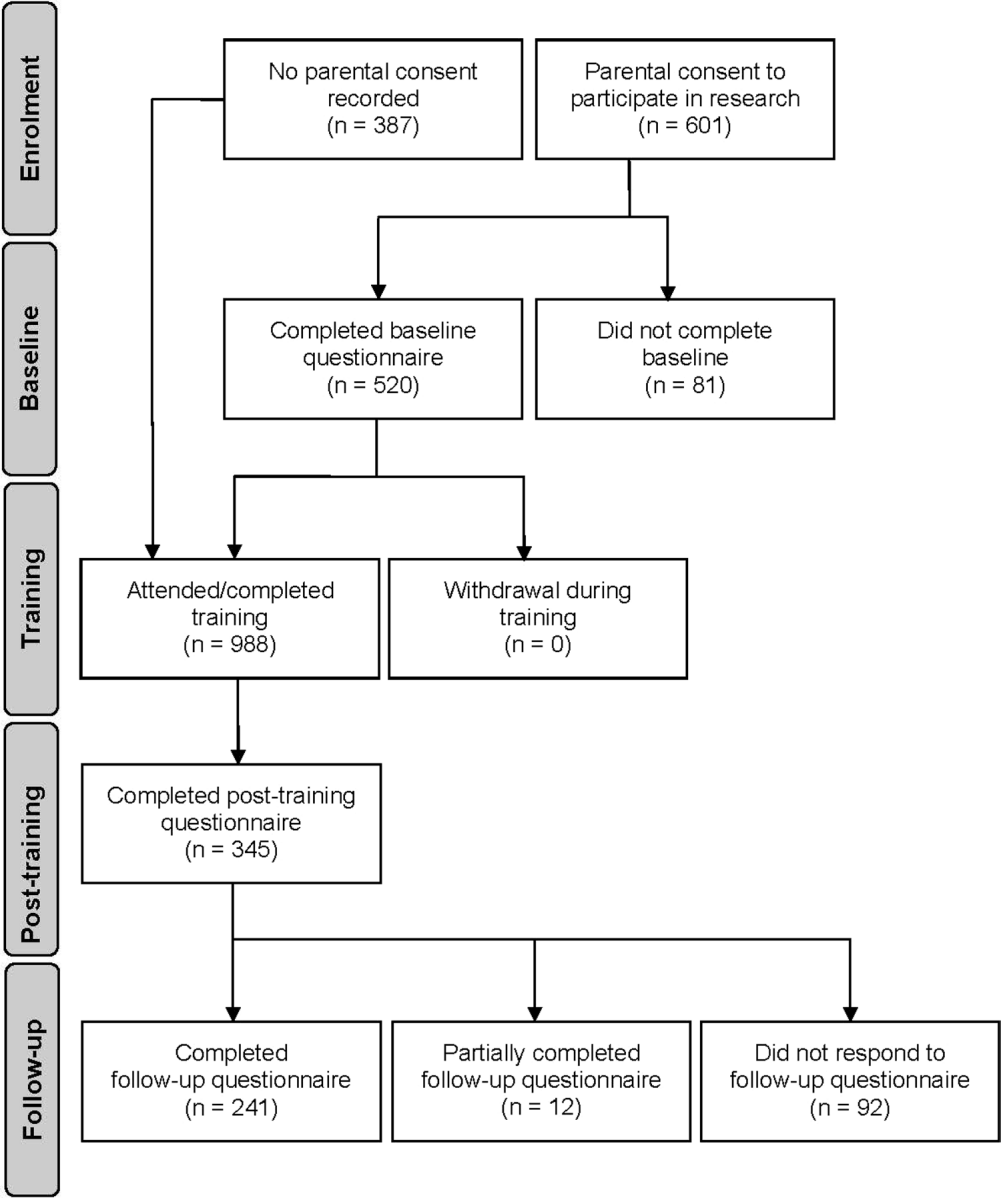
Participant flow Diagram. Note: All students in the eligible classes were offered the training, irrespective of whether they had parental consent to participate in the research or whether they completed the baseline questionnaire. 520 students completed the baseline questionnaire. 66 % of these participants went on to complete both the training and the post-training questionnaire. 253 students (49 % of baseline) completed the follow-up questionnaire 3-months after training. 12 students responded to more than 50 % but less than 100 % of the questionnaire. Participants who did not complete the post-training or follow-up questionnaires were included in the analyses using multiple imputation estimates

A diagram showing the flow of students through the stages of the evaluation research is shown in Fig. 2 [61]. The total number of eligible students who attended the training was 988, and 61 % of these had parental consent to participate in the evaluation research. From baseline to three-month follow-up, student attrition was high, with only 49 % completing the third questionnaire (46 % of males and 51 % of females). The main reason for attrition at post-training and follow-up was student non-attendance at the class scheduled for survey completion. Attrition rates differed across the four schools. Table 2 shows student participation in the repeated measures by school. School 2, the regional Catholic school, had the highest level of student participation, while the two government schools had the highest levels of student attrition.

**Table 2:** Student sample by school and measurement occasion

School	Baseline student *n*	Post-test student *n* (%) of baseline	Follow-up student *n* (%) of baseline
1. Metro independent	240	155 (65)	103 (43)
2. Regional Catholic	105	87 (83)	78 (74)
3. Metro government	106	55 (52)	45 (42)
4. Regional government	69	48 (70)	27 (39)
Total	520	345 (66)	253 (49)

### Mental health literacy

Results for *problem recognition* are shown in Table 3. This improved significantly over time for the social phobia (Jeanie) vignette, though not for the depression with suicidal thoughts (John) vignette. However, recognition was already very high at baseline, with over 85 % of students correctly reporting that John appeared to be experiencing depression.

**Table 3:** Results for the adapted Mental Health Literacy Survey for adolescents

	John vignette		Jeanie vignette	
		OR (95 % CI)/B (SE)		OR (95 % CI)/B (SE)
*Recognition of problem in vignette* ^*a*^	*% yes*		*% yes*	
Baseline	85.4		52.5	
Post-test vs baseline	83.4	0.75 (0.47–1.22)	67.5	3.99 (2.33–6.82)***
Follow-up vs baseline	87.2	0.99 (0.53–1.83)	65.3	3.11 (1.78–5.43)***
*Confidence in helping*	*%* ≥ *quite a bit*		*%* ≥ *quite a bit*	
Baseline	33.7		41.5	
Post-test vs baseline	56.6	4.41 (2.95–6.62)***	58.9	3.70 (2.46–5.56)***
Follow-up vs baseline	51.2	3.09 (2.02–4.74)***	50.6	1.76 (1.07–2.88)*
*Number of adult sources (excluding family) selected as helpful* ^*b*^				
	*M (SD)*		*M (SD)*	
Baseline	3.32 (1.60)		2.75 (1.75)	
Post-test vs baseline	4.05 (1.58)	0.70 (0.08)***	3.64 (1.81)	0.85 (0.10)***
Follow-up vs baseline	3.83 (1.66)	0.48 (0.11)***	3.62 (1.96)	0.84 (0.11)***
*Stigma: weak*-*not*-*sick*	*M (SD)*		*M (SD)*	
Baseline	1.74 (0.66)		1.92 (0.76)	
Post-test vs baseline	1.61 (0.67)	−0.12 (0.03)***	1.73 (0.78)	−0.19 (0.04)***
Follow-up vs baseline	1.66 (0.70)	−0.08 (0.04)	1.73 (0.81)	−0.17 (0.04)***
*Stigma: Dangerous and unpredictable*	*M (SD)*		*M (SD)*	
Baseline	2.84 (0.70)		1.77 (0.66)	
Post-test vs baseline	2.51 (0.87)	−0.34 (0.05)***	1.75 (0.74)	−0.03 (0.04)
Follow-up vs baseline	2.43 (0.84)	−0.44 (0.05)***	1.79 (0.82)	0.03 (0.06)
*Stigma: would not tell anyone*	*%* ≥ *disagree*		*%* ≥ *disagree*	
Baseline	46.9		56.7	
Post-test vs baseline	61.2	2.49 (1.73–3.59)***	65.2	1.72 (1.20–2.49)**
Follow-up vs baseline	54.6	1.51 (1.02–2.24)*	59.8	1.17 (0.80–1.73)
*Stigma: social distance*	*M (SD)*		*M (SD)*	
Baseline	9.85 (2.99)		8.37 (3.01)	
Post-test vs baseline	9.19 (3.12)	−0.64 (0.14)***	8.27 (3.19)	−0.08 (0.16)
Follow-up vs baseline	9.11 (3.23)	−0.64 (0.17)***	7.89 (3.12)	−0.35 (0.17)*
*Help*-*seeking intentions: talk tell ask*	*% with this response*		*% with this response*	
Baseline	14.12		9.72	
Post-test vs baseline	20.54	1.71 (1.13–2.59)*	19.94	2.45 (1.59–3.78)***
Follow-up vs baseline	22.54	2.24 (1.46–3.44)***	18.72	2.27 (1.35–3.82)**
*Help*-*seeking intentions: seek help*	*% with this response*		*% with this response*	
Baseline	77.06		74.01	
Post-test vs baseline	86.31	2.16 (1.33–3.52)**	81.87	1.79 (1.16–2.74)**
Follow-up vs baseline	84.84	1.90 (1.10–3.26)*	83.40	1.86 (1.07–3.22)*
*Help*-*seeking intentions: Non help*	*% with this response*		*% with this response*	
Baseline	25.29		24.01	
Post-test vs baseline	13.99	0.34 (0.20–0.60)***	16.01	0.48 (0.30–0.77)**
Follow-up vs baseline	13.11	0.29 (0.14–0.60)**	11.91	0.34 (0.17–0.68)**

* p < 0.05; ** p < 0.01; *** p < 0.001

^*a*^To be considered correct, responses need to mention ‘depression’ or ‘depression and suicide’. Responses mentioning suicide alone were not included

^*b*^Sources of adult help included were: Counsellor, GP, Psychologist, Teacher, School Welfare, Minister/Priest

After receiving the training, there was an increase in the number of adult sources (excluding family) that were reported as ‘helpful’ by students and this was maintained at follow-up (see Table 3). Results for *beliefs about help* from a range of different sources are shown in Table 4. Following the training, students were significantly more likely to report that a counsellor, family member, GP (family doctor), Minister/Priest, school welfare coordinator/counsellor, and teacher was likely to be ‘helpful’ for both John and Jeanie. These changes were maintained at follow-up.

**Table 4:** Beliefs about helpfulness of different sources

	John vignette		Jeanie vignette	
	*% helpful*	OR (95 % CI)	*% helpful*	OR (95 % CI)
*Close friend*				
Baseline	93.5		91.1	
Post-test vs Baseline	93.2	0.73 (0.38–1.40)	95.2	1.44 (0.74–2.78)
Follow-up vs Baseline	91.6	0.57 (0.27–1.21)	91.8	0.72 (0.35–1.49)
*Counsellor*				
Baseline	83.3		62.1	
Post-test vs Baseline	89.9	1.89 (1.14–3.14)*	79.6	3.90 (2.40–6.32)***
Follow-up vs Baseline	85.9	1.04 (0.62–1.75)	76.7	2.80 (1.62–4.83)***
*Family member*				
Baseline	71.6		69.0	
Post-test vs Baseline	82.5	2.28 (1.45–3.60)***	82.0	2.64 (1.71–4.07)***
Follow-up vs Baseline	82.3	2.26 (1.43–3.55)**	81.6	2.26 (1.36–3.75)**
*GP (Family doctor)*				
Baseline	47.4		32.8	
Post-test vs Baseline	64.2	2.83 (1.80–4.43)***	53.8	4.41 (2.88–6.78)***
Follow-up vs Baseline	57.3	2.12 (1.33–3.37)**	52.7	3.22 (1.96–5.31)***
*Minister/Priest*				
Baseline	17.8		12.1	
Post-test vs Baseline	24.3	1.98 (1.26–3.12)**	22.7	3.83 (2.28–6.45)***
Follow-up vs Baseline	24.6	2.37 (1.36–4.13)**	26.5	6.30 (3.41–11.63)***
*Parent*				
Baseline	69.6		71.5	
Post-test vs Baseline	79.3	1.70 (1.11–2.62)*	76.3	1.32 (0.88–1.98)
Follow-up vs Baseline	73.5	1.24 (0.74–2.08)	75.1	1.09 (0.66–1.82)
*Psychologist*				
Baseline	81.4		68.4	
Post-test vs Baseline	85.5	1.40 (0.86–2.26)	77.2	2.06 (1.30–3.27)**
Follow-up vs Baseline	87.5	1.78 (0.96–3.29)	78.8	2.39 (1.40–4.08)**
*School welfare coordinator/counsellor*				
Baseline	63.5		58.6	
Post-test vs Baseline	83.7	5.54 (3.35–9.16)***	75.7	3.03 (1.98–4.62)***
Follow-up vs Baseline	77.1	2.44 (1.42–4.22)**	72.2	2.33 (1.51–3.58)***
*Teacher*				
Baseline	38.7		41.3	
Post-test vs Baseline	57.1	3.55 (2.34–5.40)***	54.4	2.12 (1.44–3.10)***
Follow-up vs Baseline	49.8	2.06 (1.27–3.35)**	55.1	2.07 (1.32–3.24)**

* p < 0.05; ** p < 0.01; *** p < 0.001

### Stigmatising attitudes

Results for stigmatising attitudes are shown in Table 3. Across all four scales, there were significant improvements from baseline to post-test. These were often, though not always, maintained at follow-up. For the social phobia vignette, students were significantly less likely to believe that Jeanie was *weak*-*not*-*sick*, both after the training and at 3 months follow-up. For the depression with suicidal thoughts vignette, students were also less likely to believe John was weak-not-sick after the training, though this was not maintained at follow up. However, weak-not-sick scores for both John and Jeanie were low before the training, with means of 1.6–1.9, where a rating of 1 is equal to ‘strongly disagree’.

Scores for the *dangerous/unpredictable* scale were higher for the depression than social phobia vignette, across all measurement occasions. No significant differences were found across time for the social phobia vignette, though scores were already low. Scores for the depression vignette decreased significantly after the training and this improvement in stigma was maintained at follow-up.

For the *would not tell anyone* scale, the percentage of students who said they either strongly disagreed or disagreed significantly increased over time, suggesting that fewer students endorsed the stigmatising belief that it is better not to tell anyone about a mental health problem.

Scores on the *social distance* scale decreased over time for both the social phobia and depression vignettes, and these improvements were statistically significant at both post-test and follow-up on the depression vignette, and at follow-up for the social phobia vignette.

### Mental Health First Aid intentions and behaviours

Results for *Confidence* are shown in Table 3. More than half of all students reported feeling ‘quite a bit’ or ‘extremely’ confident in helping John/Jeanie immediately after receiving the teen MHFA training, which was a significant improvement on baseline levels. These changes were retained at three-month follow-up.

The data assessing change in quality of *MHFA intentions* were considered to be invalid after baseline and therefore not subject to analyses. At baseline 513 (depression vignette) and 510 (social phobia vignette) students provided open-ended responses to the question “If (John/Jeanie) was someone you knew and cared about, what would you do to help (him/her)?”. At post-test this was 390 (depression) and 382 (social phobia), and at follow-up 300 (depression) and 295 (social phobia). When scoring the responses against the teen MHFA action plan, it became apparent that a very large proportion of the responses at post-test and follow-up were too truncated to apply the scoring protocol appropriately. To test whether students provided increasingly truncated responses across time-points, we examined whether text length was equally distributed across all three time-points. A repeated-measures ANOVA showed that responses at baseline (*M* = 19.6 words), post-test (*M* = 8.2 words) and follow-up (*M* = 7.4 words) were significantly different, with significant truncation across time, *F* (2596) = 193.69, *p* < 0.001. While this could be a mark of more concise responses, a large proportion were considered corrupt or invalid (e.g., because they simply stated ‘no’). The decision was therefore made by the research team to consider the change data invalid.

To determine whether students were more likely to provide MHFA to a peer after receiving the training, analyses were planned to compare the number of students reporting having given first aid before and after the course, adjusting for whether students came into contact with a person requiring first aid. However, as shown in Table 5, only a small proportion of students reported having contact with a person with a mental health problem at follow-up, resulting in missing data for almost 80 % of the sample on this variable. Due to the small sample size, statistical analyses could not be conducted. Nevertheless, among these students, rates of providing MHFA were high before the training and rose following the intervention, yet it is unknown whether this change was statistically significant.

**Table 5:** Rates of mental health first aid given before and after attending the course

	% Yes	% No
*Contact in last 3* *months with a person experiencing a mental health problem or crisis*		
Baseline (n = 512)	54.3	45.7
Follow-up (n = 240)	35.8	64.2
*Help given*		
Baseline (n = 313)	87.9	12.1
Follow-up (n = 90)	91.8	8.2

To qualitatively assess the nature of first aid interactions, students who reported providing MHFA also completed the *First Aid Experiences Questionnaire* [52]. Of the 253 students who completed the follow-up questionnaire, 79 (31.2 %) provided feedback on their first aid experiences. Forced-choice responses are summarised in Tables 6 and 7. The majority of students who had an experience of providing MHFA reported believing that what they had done to assist the person was helpful and that the information in the program contributed to how helpful they were. Open-ended responses revealed that most students tried to help a friend who they thought might be experiencing depression. To help their peer, students commonly reported talking to their friend about the problem. For example, participant 28 stated “[I] talked to her about it, took her to a counsellor and had her over for plenty of sleepovers”, and participant 60 said “I told them to see a psychologist but that I would always be there for them and they could always speak to me about their feelings”. When asked whether they had done anything differently while helping their friend, since attending the teen MHFA training, participant 73 stated that “[I was] more patient and easy to deal with”. Importantly, across all responses there were no reports of negative or adverse experiences.

**Table 6:** First aid experience questionnaire for students reporting providing mental health first aid at follow-up (n = 79)

Response	N (%)
When assisting the person, did you use the information provided in the program?	
Yes	28 (35.4)
Not sure	35 (44.3)
No	16 (20.3)
Do you think what you did helped the person?	
Yes, very helpful	15 (19.0)
Yes, helpful	39 (49.4)
Neither helpful nor unhelpful	24 (30.4)
No, very unhelpful	1 (1.3)
Do you think the information in the program contributed to how helpful you were in assisting the person?	
Yes, definitely	14 (17.7)
Yes, probably	31 (39.2)
Not sure	20 (25.3)
Probably not	11 (13.9)
Definitely not	3 (3.8)
When assisting the person, did you do anything differently from what you would have done before attending the program?	
Yes	12 (15.2)
Not sure	40 (50.6)
No	27 (34.2)
Did you suggest to the person you were helping that they should talk to an adult about their problem?	
Yes	36 (45.6)
Not sure	7 (8.9)
No	4 (5.1)
N/A they were already being helped by an adult	32 (40.5)
As a result of your suggestion, did the person you were helping talk to an adult?	
Yes	22 (27.9)
Not sure	19 (24.1)
No	6 (7.6)
N/A they were already being helped by an adult	32 (40.5)

**Table 7:** Attitude changes reported by students at follow-up

Response	n (%)
In the future, if you were to come across someone who you believed was experiencing a mental health problem or crisis, how well prepared would you feel to deal with the situation? (n = 161)	
Well prepared	113 (70.2)
Neither prepared nor unprepared	11 (6.8)
Not well prepared	10 (6.2)
Unsure	2 (1.2)
Invalid	25 (15.5)
How has the “teen Mental Health First Aid” program changed how you relate to or feel about people who experience mental health problems? (n = 159)	
Better understanding of mental health problems	62 (39.0)
Gave me skills to use	29 (18.2)
Gave me more confidence in helping	10 (6.3)
Helped me relate to people with mental illness	17 (10.7)
Reinforced what I already know	17 (10.7)
No change	26 (16.4)
Invalid	16 (10.1)
Unsure	1 (0.6)

### Student mental health and help-seeking status

Results for *student mental health* are shown in Table 8. The K6 showed a significant decrease from baseline to follow-up, indicating that students were reporting less psychological distress 3 months after receiving the training. This occurred despite the third survey instrument being completed during the lead up to an exam period, which might be expected to be show an increase in K6 scores.

**Table 8:** Student mental health

Measure	M (SD)	B (SE)
*K6*		
Baseline	13.60 (5.03)	
Follow-up vs Baseline	11.92 (4.71)	−1.50 (0.28)***
*Self*-*report*	Baseline n (%)	Follow-up n (%)
Yes	108 (21)	48 (19)
Not sure	87 (16)	35 (14)
No	318 (63)	169 (67)

*** p < 0.001

Results for *Student help*-*seeking intentions* are shown in Table 3. Over time, students were significantly more likely to report that they would talk, tell or ask someone if they had a problem like John’s or Jeanie’s, significantly more likely to report some form of help-seeking, and significantly less likely to report a non-help-seeking action, such as pushing people away or doing nothing.

Students’ *self*-*report of mental health problems or crises* revealed that the proportion of students reporting a problem at baseline was greater than the proportion of students reporting a problem at follow-up (see Table 8).

**Fig. 3:**
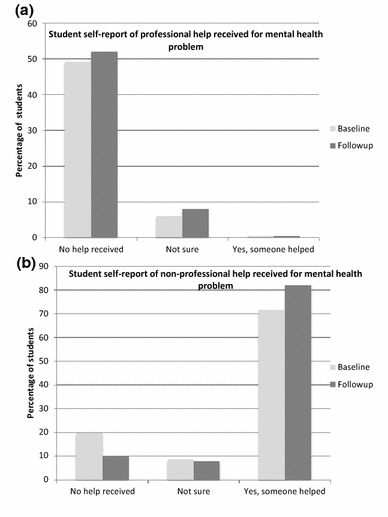
Student self-report of professional and non-professional help received for a mental health problem. Note: 108 students reported having a mental health problem in the 3 months before receiving teen MHFA. 48 students reported having one in the 3 months after the course. Of those students, the majority reported not having received professional help (**a**), but did report having received help from someone else, such as a teacher, parent or friend (**b**)

Given the small number of students reporting a mental health problem at follow-up, statistical analyses of any changes over time in *Self*-*report of help received* could not be conducted. A greater proportion of students reported receiving help from a non-health professional source (e.g. a teacher, parent or friend), compared to receiving help from a health professional, at both baseline and follow-up (see Fig. 3a). A large majority of students reported not having received professional help at both baseline and follow-up (see Fig. 3b).

### Participant satisfaction

**Fig. 4:**
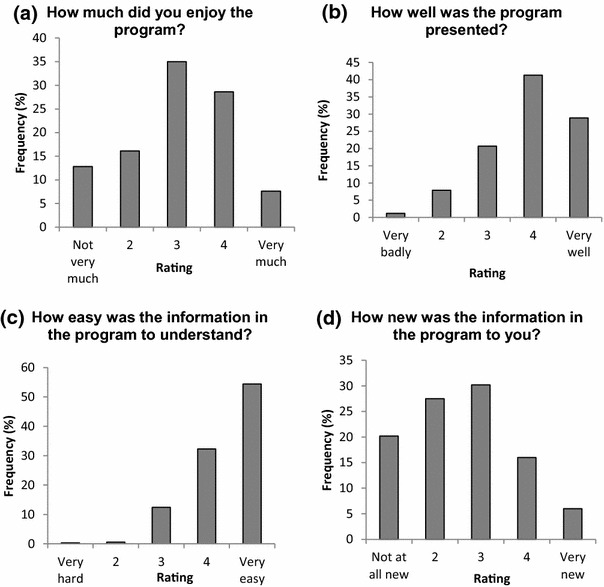
Participant satisfaction with the teen MHFA Program. Note: 331 students completed the post-training questionnaire which included questions about participant satisfaction with the program content and presentation. **a** shows that a majority of students reported enjoying the program. **b** shows a majority rated the program as well presented. **c** shows the majority thought the program was very easy to understand. **d** shows that most students reported the program content was not new to them

**Fig. 5:**
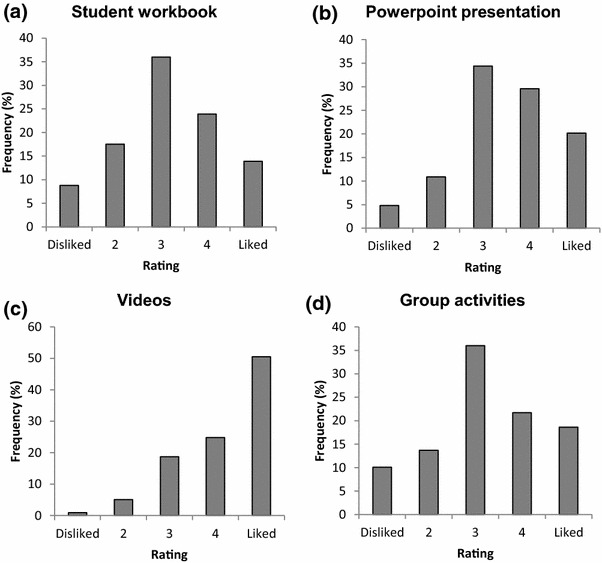
Participant satisfaction with the teen MHFA materials. Note: 331 students completed the post-training questionnaire which included questions about participant satisfaction with the course materials. **a** shows that a majority of students reported liking the student workbook. **b** shows a majority also liked the powerpoint presentation. **c** shows that the videos were very well liked among the students and clearly the most popular aspect of the course. **d** shows that most students reported liking the group activities

Students also completed a brief *Participant Satisfaction Questionnaire* as part of the post-intervention survey. Results are shown in Figs. 4 and 5. The majority of students thought the program was easy to understand, very well presented and enjoyable. The videos were the most liked materials of the course, with the Powerpoint presentation also being well rated.

Seventy-six adults completed the *Teacher and ParentSatisfaction Questionnaire.* They reported having a good knowledge of the content that students had been taught and that a majority of students thought the program was enjoyable, well presented and useful for the students.

## Discussion

The aim of the current study was to conduct an uncontrolled pilot evaluation of teen MHFA to provide initial evidence of program effects, test relevant measures of student knowledge, attitudes and behaviours, and to examine the feasibility of providing the program in Australian secondary schools. Although there was a large number of students who did not complete the three-month follow-up assessment, which left some planned statistical analyses too underpowered to be conducted, the conservative analyses did reveal that the program was associated with statistically significant improvements in mental health literacy, decreases in stigmatising attitudes, greater confidence in providing MHFA to a peer, increases in intention to seek help and decreases in reporting of psychological distress.

### Evidence of program effects

Results for the measures of mental health literacy and stigmatising attitudes appear particularly promising. Given that teen MHFA is a fairly simple 3.75 h intervention, it is encouraging to see that, after receiving the course, students were more than five times more likely to consider a school counsellor as helpful for a young person with a mental health problem, and more than two times more likely to reject the stigmatising belief that you should not tell anyone about a mental health problem. Students were also four times more likely to report feeling confident in helping a peer with a mental health problem after receiving the teen MHFA training. Given that confidence has been found to be a predictor of quality first aid intentions [30, 51, 62–66], this finding is particularly important. This is also reflected in the large number of students who reported feeling ‘well prepared’ for helping someone in the future if they were to come across a peer with a mental health problem, even though they had not experienced a first aid situation in the last 3 months.

Despite the lack of statistically significant results arising from measures of MHFA behaviours, the qualitative data collected in the *First Aid Experiences Questionnaire* were devoid of any negative experiences described by students in trying to assist a peer with a mental health problem or crisis. It therefore appears that the teen MHFA training did not have any negative impact on first aid behaviours.

It is also encouraging that the measure of psychological distress provided no evidence that the course was associated with any iatrogenic effects on student mental health. Indeed, reports of psychological distress decreased from baseline to follow-up. While a previous evaluation of MHFA found a statistically significant positive impact on participant mental health [67], this has not been consistently found [68]. It is possible that this finding is a re-test effect, which is often found on such measures [69]. Similarly, students’ self-report of mental health problems or crises showed a decrease from baseline to follow-up. However, given that self-report of a mental health problem was a predictor of drop-out, it is possible that students with mental health problems were less likely to engage in the training and research program or were not present for the administration of the final questionnaire, given higher rates of absenteeism and truancy in students with mental illness [70]. These findings indicate that further evaluation of teen MHFA using a randomised controlled trial design would be valuable in elucidating whether the program is benefitting student mental health.

Importantly, although the numbers of students reporting a mental health problem were too small to be subject to statistical analyses, help seeking intentions were assessed by asking students if they had a problem like John’s/Jeanie’s what they would do about it. All analyses revealed statistically significant improvements after receiving the teen MHFA training, and that these improvements were maintained 3 months later.

### Limitations

Two major limitations of the current study are the participant drop-out rate and the high rate of non-responses to a number of important open-ended questions. Because a large proportion of the student sample did not complete the follow-up questionnaire, the resulting small sample hampered statistical analyses and the findings relating to any significant changes in MHFA behaviours remain ambiguous. In particular, the measurement of MHFA behaviours relied heavily on open-ended responses, which were not well received by the student participants. That students’ open-ended responses became significantly truncated over time is an important finding of this study, and has provided a valuable lesson in crafting appropriate measurement instruments for future evaluations of MHFA training with high-school students.

Previous research into question format has found that students perform better on open-ended questions if they have already answered structured (i.e., multiple choice) versions first [71]. In future trials, forced-choice response options will therefore be offered (using the most common responses from the current study), alongside a free-text ‘other’ option, to capture any ideas that are not covered by the given response set. We believe that this alternative response format will be a much more acceptable form of gathering responses from young people.

### Feasibility of providing the program in Australian secondary schools

Results from the participant satisfaction surveys revealed that students largely enjoyed the teen MHFA training, found it useful and engaging. A minority rated the content as not new to them, perhaps reflecting exposure to other mental health promotion programs available in Australia and to the popularity of psychology as a high school subject. The most highly rated aspect of the program was the videos. These findings were complemented by the positive reactions of parents and teachers to the program, who also reported that they thought teen MHFA was useful and enjoyable for students. Therefore, despite a clear and frank discussion of suicidal ideation in secondary school classrooms, the teen MHFA training program appears to be well received by students and adults. Furthermore, this evaluation found no evidence of any iatrogenic effects on student psychological distress or negative first aid outcomes.

### Study significance

As our understanding of the importance of improving adolescent mental health literacy and help-seeking has improved, a number of education programs have been developed for adolescents within secondary schools [72–75]. Two innovative aspects of the teen MHFA program are that it is provided by a trained instructor with experience in adolescent mental health, and focuses on developing first aid behaviours to help a peer. Although other programs have been designed to improve mental health literacy and help-seeking, they do not address the issue of peer-to-peer disclosure of mental health problems, which appears to be an initial step on the help-seeking pathway for many adolescents, especially girls [22]. Most recently, another Australian school program, *headstrong*, was evaluated using a cluster randomised controlled trial, with 380 year 9 and 10 students across 10 Catholic and Independent schools [76]. This program consists of 10 h of content delivered by the regular classroom teacher, and is designed to increase mental health literacy for depression, decrease stigmatising attitudes and improve help-seeking in adolescents with mental health problems. Although all measures except those used to assess stigmatising attitudes were different, the results provide a point of comparison to the findings of the current study. A significant group (*headstrong* program vs normal classroom control) by time (pre-, post and 6-month follow-up) interaction was found for the *dangerous/unpredictable* scale, but not the *weak*-*not*-*sick*. For the latter, there was a main effect of time, indicating that both groups reduced in their belief that adolescents with depression are ‘weak’ not ‘sick’ across measurement occasions. This may indicate that the current findings are re-test effects, rather than a result of the teen MFHA program. However, the *headstrong* evaluation failed to find any statistically significant improvements on measures of help-seeking or student mental health, across both the intervention and control groups. Indeed, improvements in help-seeking intentions have been found in only one other program evaluation [72] and appear to be a particularly difficult factor to improve. Although the current results come from an uncontrolled pilot trial, significant improvements were seen for psychological distress and help-seeking intentions, suggesting that teen MHFA may have important differences to previous programs. Certainly the results of the current study indicate that a randomised control trial is warranted, to further understand the causal effects of the teen MHFA training on these important outcomes for adolescents.

## Conclusions

Although a large proportion of students failed to participate in post-training assessments, the conservative analyses conducted revealed that the teen MHFA program appears to be associated with statistically significant improvements in mental health literacy, decreases in stigmatising attitudes, confidence in providing MHFA to a peer, increases in intentions to seek help and improved student mental health. The program is feasible for delivery to secondary school students and further research using refined measures, better suited to students, is indicated.

## Additional files

10.1186/s13033-016-0034-1 Sociodemographic characteristics of the four schools in 2013.
10.1186/s13033-016-0034-1 Vignettes. An abbreviated Mental Health Literacy Interview, adapted for use in written form, was used to assess knowledge, beliefs and first aid behavioural intentions. This involved presentation of two vignettes; one depicting an adolescent experiencing depression with suicidal ideation (John), and another experiencing social phobia (Jeanie).
10.1186/s13033-016-0034-1 Teacher and Parent Satisfaction Questionnaire. A brief questionnaire was offered to parents and teachers of students who attended the teen MHFA training. The online questionnaire was administered 3 months after students had received the training and designed to qualitatively examine adults’ perceptions of the course and students’ training experience. A table summarizing results from the 76 participants who completed the questionnaire are shown in Additional file 2.
