# Supplementary material for: ‘teen Mental Health First Aid’: a description of the program and an initial evaluation
Source: Int J Ment Health Syst. 2016 Jan 19;10:3. doi: 10.1186/s13033-016-0034-1 (PMC4717562; doi:10.1186/s13033-016-0034-1)
Supplement: Supplementary file 1 — 10.1186/s13033-016-0034-1 Sociodemographic characteristics of the four schools in 2013. [file 13033_2016_34_MOESM1_ESM.docx]

# Additional file 1

**Sociodemographic characteristics of the four schools in 2013**

| Characteristic | School 1 | School 2 | School 3 | School 4 |
| --- | --- | --- | --- | --- |
| Location | Metropolitan | Provincial | Metropolitan | Provincial |
| School sector | Non-government | Non-government | Government | Government |
| Year range | Prep-12 | 7-12 | 7-12 | 7-12 |
| ICSEA* | 1171 | 1061 | 1143 | 1009 |
| Total enrolments | 2927 | 791 | 834 | 1055 |
| Percent girls | 42 | 52 | 48 | 51 |
| Percent language other than English | 17 | 6 | 14 | 4 |
| Percent Indigenous | 0 | 0 | 1 | 1 |

* ICSEA = Index of Socio-Educational Advantage, with M =1000 and SD =100
