# Supplementary material for: ‘teen Mental Health First Aid’: a description of the program and an initial evaluation
Source: Int J Ment Health Syst. 2016 Jan 19;10:3. doi: 10.1186/s13033-016-0034-1 (PMC4717562; doi:10.1186/s13033-016-0034-1)
Supplement: Supplementary file 3 — 10.1186/s13033-016-0034-1 Teacher and Parent Satisfaction Questionnaire. A brief questionnaire was offered to parents and teachers of students who attended the teen MHFA training. The online questionnaire was administered 3 months after students had received the training and designed to qualitatively examine adults’ perceptions of the course and students’ training experience. A table summarizing results from the 76 participants who completed the questionnaire are shown in Additional file 2. [file 13033_2016_34_MOESM3_ESM.docx]

# Additional file 3

### Teacher and Parent Satisfaction Questionnaire (n = 76)

n **(%)**

*Gender*

Female 68 (89.5)

Male 8 (10.5)

*Involvement with the teen MHFA program*

Parent of student who received the program 71 (93.4)

Teacher 4 (5.3)

Welfare coordinator 1 (1.3)

*Have you ever attended a Mental Health First Aid course (e.g. Youth, Standard, Aboriginal and Torres Strait Islander Mental Health First Aid)?*

Yes 22 (29.0)

No 52 (68.4)

Not sure 2 (2.6)

*Have you spoken to the students/your child about the program and its contents?*

Yes 60 (89.6)

No 7 (10.5)

*Do you feel like you have a good understanding of what was presented to and discussed with the students/your child?*

Yes 40 (59.7)

No 15 (22.4)

Not sure 12 (17.9)

*How do you think the students/your child responded to the program?*

Very negatively 0 (0.0)

2 3 (4.5)

3 12 (17.9)

4 26 (38.8)

Very positively 18 (26.9)

Not sure 8 (11.9)

*How useful do you think the information in the program was for the students/your child?*

Not at all useful 0 (0.0 )

2 4 (6.0)

3 6 (9.0)

4 29 (43.3)

Very useful 21 (31.3)

Not sure 7 (10.5)

*How well do you think the program was presented to the students/your child?*

Very badly 0 (0.0)

2 4 (6.0)

3 6 (9.0)

4 25 (37.3)

Very well 18 (26.9)

Not sure 14 (20.9)

*How much do you think the students/your child enjoyed the program?*

Not very much 4 (6.0)

2 4 (6.0)

3 17 (25.4)

4 24 (35.8)

Very much 8 (11.9)

Not sure 10 (14.9)
